# Efficacy of a WeChat-Based, Multidisciplinary, Full-Course Nutritional Management Program on the Nutritional Status of Patients With Ovarian Cancer Undergoing Chemotherapy: Randomized Controlled Trial

**DOI:** 10.2196/56475

**Published:** 2024-11-04

**Authors:** Xiaojuan Tian, Yan Liu, Jiahua Zhang, Lixiao Yang, Linyao Feng, Aidong Qi, Hanjiazi Liu, Pengju Liu, Ying Li

**Affiliations:** 1 Ward 2, Gynaecological Oncology Centre, Peking Union Medical College Hospital, Chinese Academy of Medical Sciences, Beijing, China; 2 Department of Nutrition, Peking Union Medical College Hospital, Chinese Academy of Medical Sciences, Beijing, China; 3 Ward 1, Department of Obstetrics, Peking Union Medical College Hospital, Chinese Academy of Medical Sciences, No.1 Shuaifuyuan, Dongcheng District, Beijing, 100730, China, 86 13582506099

**Keywords:** WeChat, nutrition management, ovarian cancer, chemotherapy, mobile health

## Abstract

**Background:**

As the most malignant type of cancer in the female reproductive system, ovarian cancer (OC) has become the second leading cause of death among Chinese women. Chemotherapy is the main treatment for patients with OC, and its numerous adverse effects can easily lead to malnutrition. It is difficult to centrally manage patients with OC in the intervals between chemotherapy. The use of WeChat, an effective mobile tool, in chronic disease management has been highlighted.

**Objective:**

This study aimed to implement a continuous follow-up strategy and health monitoring based on the WeChat platform for patients with OC undergoing chemotherapy to ensure that each phase of chemotherapy was delivered on schedule and to improve the survival rate of patients with OC.

**Methods:**

Participants were recruited and randomly assigned to either the WeChat-based nutrition intervention group or the usual care group. A self-administered general information questionnaire was used at enrollment to obtain basic information about the patients. The Patient-Generated Subjective Global Assessment (PG-SGA) Scale was used to investigate the nutritional status of the patients at 3 time points (T0=before the first admission to the hospital for chemotherapy, T1=2 weeks after the first chemotherapy, and T6=2 weeks after the sixth chemotherapy). The blood indices of patients were investigated through the inhospital health care system at 3 times（T0=before the first admission to the hospital for chemotherapy, T1=2 weeks after the first chemotherapy, and T6=2 weeks after the sixth chemotherapy). Patients in the intervention group were introduced to the nutrition applet, invited to join the nutrition management group chat, and allowed to consult on nutritional issues in private chats with nutrition management team members. Linear mixed models were used to analyze changes in each nutritional indicator in the 2 groups, with their baseline measurements as covariates; with group, time, and group-time interactions considered as fixed effects; and with patients considered as random effects.

**Results:**

A total of 96 patients with OC undergoing chemotherapy were recruited into the study. Distribution was based on a 1:1 ratio, with 48 patients each in the nutrition intervention group and the usual care group. The attrition rate after the first chemotherapy session was 18.75%. The mixed linear model revealed that the group-based effect and the group-time interaction effect on PG-SGA scores were significant (*F*_38,38_=4.763, *P*=.03; *F*_37,37_=6.368, *P*=.01), whereas the time-based effect on PG-SGA scores was not (*F*_38,38_=0.377; *P*=.54). The findings indicated that the group-based effect, the time-based effect, and the group-time interaction effect on nutrition-inflammation composite indices were significant (*F*_38,38_=7.653, *P*=.006; *F*_38,38_=13.309, *P*<.001; *F*_37,37_=92.304, *P*<.001; *F*_37,38_=110.675, *P*<.001; *F*_38,38_=10.379, *P*=.002; and *F*_37,37_=5.289, *P*=.02).

**Conclusions:**

This study provided evidence that a WeChat-based, multidisciplinary, full-course nutritional management program can significantly improve the nutritional status of patients with OC during chemotherapy.

## Introduction

Ovarian cancer (OC) is the most malignant type of tumor in the female reproductive system, with a poor prognosis [1]. According to the latest statistics, there are 196,000 estimated OC cases, 45,000 estimated new cases, and 29,000 estimated OC deaths in China, making this disease become the second leading cause of death among Chinese women [2].

Approximately 90% of patients with OC receive chemotherapy. The carboplatin-paclitaxel combination as the first-line chemotherapy regimen for OC has shown considerable efficacy over the past 30 years [3]. Unfortunately, one of the major adverse effects of chemotherapy is malnutrition. Malnutrition is defined as a nutritional condition in which deficiencies of energy, protein, and other nutrients have measurable adverse effects on tissue or body form, function, and clinical outcomes [4]. The most common adverse effects of chemotherapy, including anorexia, altered taste and smell, food aversions, nausea and vomiting, mucositis, xerostomia, constipation, diarrhea, and early satiety, negatively affect nutritional status [5,6]. Malnutrition has been shown to be one of the leading causes of death in patients with OC [7-9]. It not only severely diminishes the efficacy of treatment but also leads to increased complications, decreased quality of life, prolonged hospitalization, increased health care costs, and shorter survival time [10]. Malnutrition may occur at any time during the perichemotherapy period, making it necessary to manage nutrition throughout the entire course of chemotherapy for patients with OC.

Surprisingly, there is less information available on nutritional management for patients with OC undergoing chemotherapy. Nutritional interventions in previous studies recommended to increase protein and calorie intake [11,12] or to modify the structure of the diet by adopting a Mediterranean diet [11]. The form of intervention was mainly counseled with a dietitian every 3 weeks through telephone [11]. The previous studies lacked real-time monitoring and timely guidance for patients, and there has been a lag in the nutritional management. In addition, they mentioned only guidance on energy intake and dietary patterns and lacked targeted nutritional guidance for chemotherapy-related adverse effects. The guideline provided more detailed dietary guidance for patients with OC undergoing chemotherapy, such as not using restrictive energy intake diets [13,14] and taking oral nutritional supplements according to the actual situation [15,16], which can help improve the nutritional knowledge of patients with OC. However, it did not mention in what way (eg, online or offline), and at what frequency and intensity, the intervention for patients treated with chemotherapy for OC is more conducive to their nutritional management. In addition, the difficulty of centrally managing patients recuperating at home between chemotherapy treatments should also be considered. Personalized nutritional guidance and education are the preferred methods of nutritional intervention for patients with OC treated with chemotherapy and should be carried out throughout the consultation [13]. Therefore, the construction of personalized nutritional management programs and the development of innovative telemedicine interventions for patients with OC undergoing chemotherapy have become urgent issues.

WeChat, a very popular social application in China, has more than 1 billion monthly active users. It is easy to operate and offers multiple functions such as text and voice messaging, free voice and video calls, group chats, subscriptions to public accounts and applets, and so forth. WeChat has been demonstrated to be an effective and more cost-efficient technological tool for chronic disease management [17,18]. Currently, the applications of WeChat in patients with cancer mainly focus on discharge follow-up [19], symptom management [20,21], cancer prevention [22], and intervention of psychological problems [23]. The effect of WeChat application in the nutritional management of patients with cancer has not been explored. Therefore, the aim of this study was to implement a continuous follow-up strategy and health monitoring based on a WeChat platform for patients with OC undergoing chemotherapy to ensure that each phase of chemotherapy was delivered on schedule and to improve the survival rate of patients with OC.

## Methods

### Study Design

We conducted a parallel, 2-armed, open-label, randomized controlled trial to examine the efficacy of the WeChat-based nutrition intervention compared with usual nutritional care and to explore preliminary effects of the intervention. The study was conducted between February 2023 and October 2023 at Peking Union Medical College Hospital, Chinese Academy of Medical Sciences in Beijing, China.

### Participants

Patients were eligible if they were aged 18 years or older with pathologically confirmed OC [24]. The chemotherapy regimen was paclitaxel in combination with carboplatin. Patients had to have normal cognitive ability and proficiency in the use of WeChat. Patients were excluded if they had a malignant tumor of another system, a serious illness, or failure of vital organs (eg, the heart, lungs, liver, and kidneys) or if they were receiving enteral or parenteral nutritional support. All patients provided written informed consent (Multimedia Appendix 1).

### Randomization and Allocation

Patients were randomly assigned (1:1) to receive the WeChat-based remote nutrition intervention or usual nutritional care. Randomization was stratified by disease stage (stages I and II vs stages III and IV) and surgical procedure (laparoscopy vs laparotomy) to balance treatment assignment. The random allocation sequence was generated by the study designers (XJT and YL, who were not involved in patient enrollment) by means of a table of random numbers, and the allocation was hidden by the envelope.

### Study Procedure

#### Intervention Group

##### Establishment of Nutrition Management Team

The nutrition management team consisted of a nursing administrator (bachelor’s degree, associate nurse practitioner), 2 gynecologic oncologists (PhD, attending physician), 2 oncology specialist nurses (bachelor’s degree, charge nurse practitioner), 1 nutrition specialist nurse (bachelor’s degree, charge nurse practitioner), 1 dietitian (PhD, associate physician practitioner), and 2 master’s degree students in nursing.

##### Design of the WeChat Platform

###### Overview

**Figure 1.**
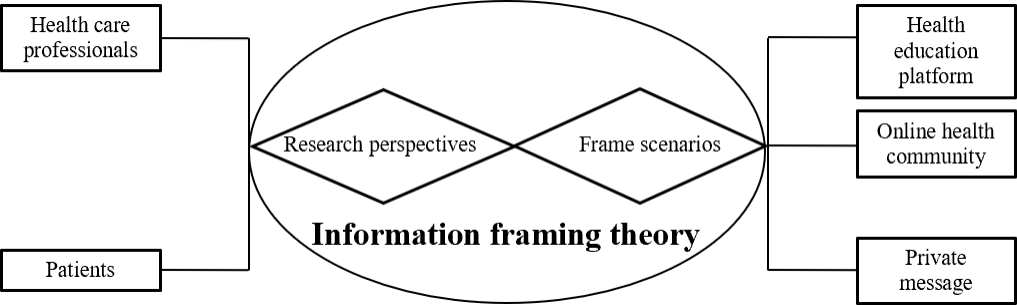
Information framing theory diagram.

The construction of the WeChat platform relied on information framing theory [25], which emphasized the impact of information on human health behavioral decision-making and health outcomes. It focused the interaction process between people and information on the manifestation of information itself, while reinforcing the dominance of information in both research perspectives and research contexts. The WeChat platform constructed in this study considered the perspectives of both health care professionals and patients and set up 3 different scenarios based on the different needs of the 2 subjects: a health education platform (WeChat applet), a web-based health community (WeChat group chat), and a private question-and-answer session (WeChat private message), with the aim of realizing the maximum benefits of nutritional management. Specific relationships are displayed in Figure 1.

###### WeChat Applet

**Figure 2.**
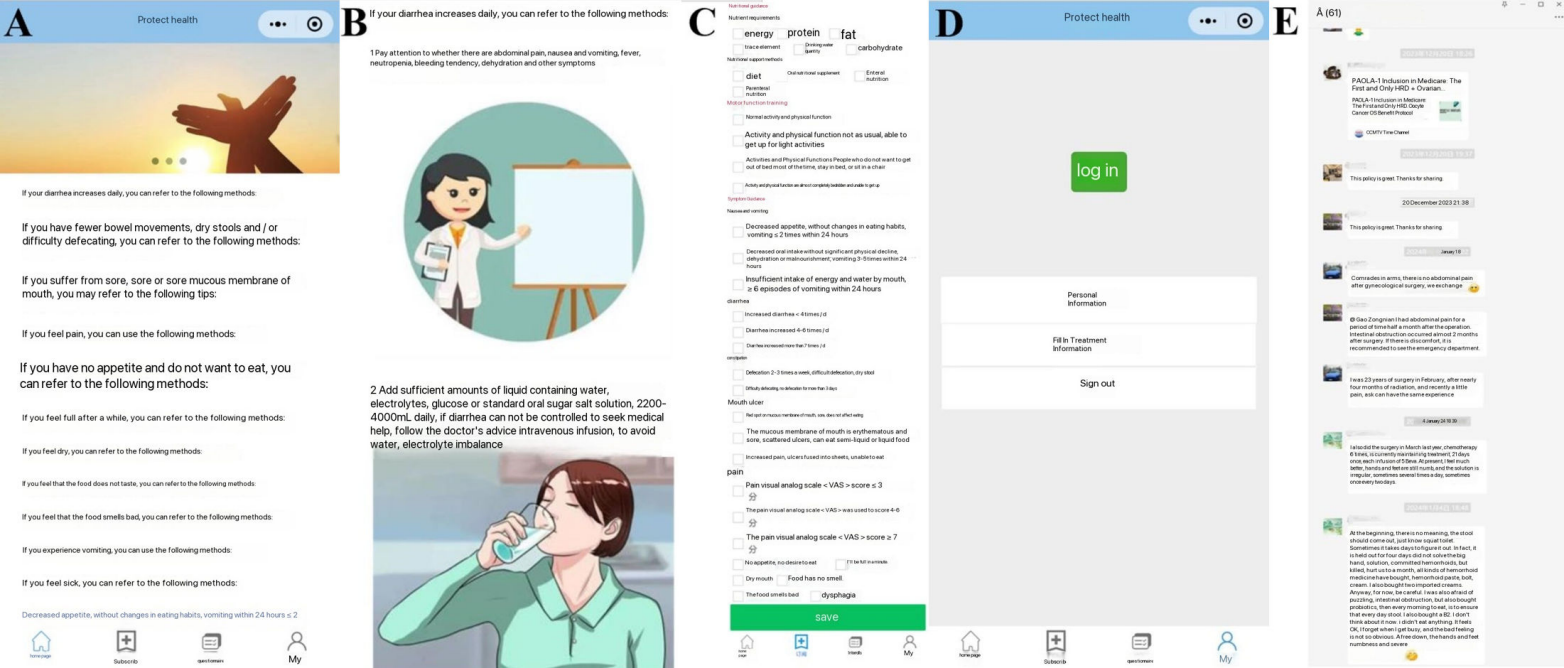
WeChat applet interface settings. (A) The applet was set up with 4 modules, namely, health education column, subscription content column, questionnaire column, and personal information column. (B) The content of the health education column. (C) The subscription content section summarized the 3 main sections of instructions: nutritional guidance, motor function training, and symptom guidance. (D) The personal information section was simple to set up and included basic patient information, treatment information, and an option to log out. (E) A group chat for patient-directed learning and encouraging patients to ask questions, share personal experiences, and discuss lifestyle topics.

With regard to the development of the nutrition WeChat applet “Good Nutrition,” it consisted of 2 ports: the computer side and the WeChat side. The computer side was operated and managed by health professionals. The WeChat terminal could be operated by patients with OC undergoing chemotherapy. Patient privacy and data security were protected through access control and permission control, patient data transfer and anonymization, redundant storage, and data backup. The applet was set up with 4 modules: health education column, subscription content column, questionnaire column, and personal information column (Figure 2A).

The content of the health education column was compiled based on a previously constructed multidisciplinary full-course nutritional management program (Table S1 in Multimedia Appendix 2) for patients with OC during chemotherapy, which mainly included chemotherapy symptom management (eg, diarrhea, nausea and vomiting, mouth ulcers), nutritional risk management (based on the Patient-Generated Subjective Global Assessment [PG-SGA] score), and physical activity management. This section provided comprehensive management measures based on symptom severity rating, nutritional risk level, and activity level. In addition, we used a combination of text and images in the presentation of the content, making it detailed and easy to understand (Figure 2B).

The subscription content section summarized the 3 main sections of instructions: nutritional guidance (nutrient requirements and nutritional support methods), motor function training, and symptom guidance (nausea, vomiting, diarrhea, constipation, mouth ulcers, and pain). On the one hand, patients could subscribe to the content they were interested in according to their own situation, and the system would remind them to check whether the subscription content has been updated. On the other hand, members of the nutrition team could obtain the subscription status of the patients from the back end, so as to have a better understanding of the adverse effects of the individuals during chemotherapy and thus give more targeted guidance (Figure 2C).

Patients were briefly quizzed on a regular basis in the form of a questionnaire, and open-ended questions were included to ask whether they had any questions about the nutritional management process.

With regard to the log-in interface, the personal information section was simple to set up and included basic patient information, treatment information, and an option to log out (Figure 2D).

###### Group Chat

Throughout the intervention phase, we sent out evidence-based nutrition papers taken from WeChat applets in group chats for patient-directed learning and encouraged patients to ask questions, shared personal experiences, and discussed lifestyle topics. We also critically analyzed the public papers shared by patients to help them judge the quality of nutritional information (Figure 2E).

###### Private Message

Patients can always choose to have a private chat with a member of the nutrition management team via WeChat. For example, a patient with an aggravated chemotherapy reaction may seek help in a private chat, and our doctors would give some professional advice. In addition, for common problems, we also posted answers in the group for other patients’ reference.

### Implementation Procedure

Detailed and clear interventions are shown in Table 1.

**Table 1.** Interventions.

Time of intervention	Objectives of intervention	Interventions
1. The first admission for chemotherapy	To enable patients to master the nutrition management WeChat platform (WeChat applet, WeChat group chat, WeChat private message) function and operation use method	WeChat applet: Searched for the “Good Nutrition” WeChat applet Added it to My Applet Opened the notification permission Logged in the Applet Introduced to the main functional sections Group chat: Invited to join the group chat Informed that throughout the intervention phase, evidence-based scientific papers would be sent to the group chat. Asking questions, sharing personal experiences, and discussing lifestyles were permitted. Group chat could help identify questionable nutrition information. Answer questions via private message: Allowed private chats with nutrition team members if in doubt.
2. During each session of chemotherapy	Enable patients to understand in advance the possible adverse reactions related to chemotherapy and the countermeasures	Explained the complications associated with chemotherapy, and given a chemotherapy care booklet and a leaflet on diet and nutrition. Before discharge, the patients were given the nutritional guidebook again.
3. Each chemotherapy interval	Real-time monitoring of patients’ chemotherapy adverse reactions and nutritional status, and timely resolution of patients’ dilemmas in coping with chemotherapy-related adverse reactions	WeChat applet: Updated the health education content every 2 months. Checked the top 3 health education topics in the subscription content weekly, and sent relevant papers to the group chat for discussion and analysis. Pushed the nutritional knowledge questionnaire weekly, according to the patients’ answer situation in the group chat for nutritional guidance. Group chat: Summarized nutritional knowledge questions once a week and answered centrally. Summarized and corrected the misconceptions shared by patients during the day every night. Answer questions via private message: One nutrition team member would be responsible for answering questions via private message and replying promptly after seeing the message every day.

### Control Group

For patients in the usual care group, upon admission, patients were provided with admission counseling, given an explanation about the complications associated with chemotherapy, and given a chemotherapy care booklet and a leaflet on diet and nutrition. Before discharge, the patients were given the nutritional guidebook again. Responsible nurses made 1 telephone follow-up visit between chemotherapy sessions.

### Data Collection and Outcomes

Self-reported sociodemographic characteristics (eg, age, BMI, marital status, education level, etc) and clinical disease features (eg, disease stage, surgical procedure, etc) were collected at baseline.

The primary outcome was assessed by the PG-SGA Scale to evaluate the nutritional status of patients at 3 time points (T0=before the first admission to the hospital for chemotherapy, T1=2 weeks after the first chemotherapy, and T6=2 weeks after the sixth chemotherapy). The PG-SGA was used as a prognostic tool developed specifically for patients with cancer to evaluate the nutritional status [26]. It consisted of 2 subscales: the patient self-assessment scale and the medical staff assessment scale. The former integrated short-term weight loss, food intake (including amount eaten, type of food, manner of eating, etc), symptoms affecting eating, and activity or physical functioning. The latter included medical history, metabolic stress, and physical examination provided by medical staff [27]. Each item in the PG-SGA Scale has a score range of 0‐4. The more severe the symptoms in relation to malnutrition, the higher the assigned value. The total score was compared and analyzed in three groups: (1) well nourished (0‐1 point), (2) moderately or suspected of being malnourished (2‐8 point), and (3) severely malnourished (≥9 point) [28]. A total score between 0 and 35 quantitatively informs the severity of malnutrition and types of intervention needed: 0‐1 point indicates no need for any intervention, 2‐3 points suggest education needs for the patients and the family, 4‐8 points indicate the need for a referral to a dietitian, and a score of 9 or more recommends an action of critical nutritional intervention. The PG-SGA Scale had high validity (sensitivity 100%, specificity 88%) [29]. It had been accepted by the Oncology Nutrition Dietetic Practice Group of the American Dietetic Association as the standard for nutrition assessment for patients with cancer [26,30,31].

The secondary outcome was to compare the blood test indices of the 2 groups at the same 3 time points, including nutrition-related blood indices, such as total protein (g/L), albumin (g/L), prealbumin (g/L), and hemoglobin (g/L); inflammation-related blood indices, such as leukocytes (10^9^/L), lymphocytes (10^9^/L), neutrophils (10^9^/L), and platelets (10^9^/L); and nutrition-inflammation composite indices, such as the prognostic nutritional index (PNI) and systemic immunoinflammatory index (SII). The PNI reflected the chronic inflammation, immune status, and nutritional status of patients with cancer and can be used to predict the risk of postchemotherapy complications in patients with cancer. The PNI  is calculated as “albumin (g/L) + 5 × lymphocyte (10^9^/L)” [32]. The SII is a new scoring system proposed by Chinese scholars in 2014 to assess the functional status of the immune system in patients with tumor [33]. The SII is calculated as “platelet (10^9^/L) × neutrophil (10^9^/L)]/lymphocyte (10^9^/L),” and the composite score combining the 3 indexes can better reflect the balance of the body’s inflammatory response and immune status. The nutrition-related blood indices (total protein, albumin, prealbumin, and hemoglobin), inflammation-related blood indices (leukocytes, lymphocytes, neutrophils, and platelets), and nutrition-inflammation composite indices (PNI and SII) were collected from hospital electronic medical records.

Patient enrollment and baseline information collection were done by the member of the group responsible for recruitment, who was aware only of the patient inclusion and exclusion criteria and was not aware of the patient grouping. The assessment of the outcome indicators—PG-SGA and blood test indices—was done by the member responsible for efficacy assessment, who was also unaware of the patient subgroups. This study was open-labeled.

### Statistical Analysis

Based on the primary outcome of PG-SGA score, a target sample size of 78 participants was estimated to provide 90% power with an α value of .05 to detect a difference between control and intervention groups. This calculation was calculated using PASS 15.0 (NCSS LLC), referring to data from previous study [34], taking into account a 20% dropout rate. Baseline characteristics were presented as medians (IQRs) or percentages and were compared using the chi-square test or the Mann-Whitney test, as appropriate. A linear mixed model was used to analyze these score changes, with their baseline measurements used as covariates. Group, time, and group-time interaction were treated as fixed effects, while the patient was treated as a random effect. Missing data due to loss to follow-up were assumed to be missing at random. The estimated within- and between-group differences were reported with their respective 95% CIs. A 2-sided *P* value of <.05 was considered statistically significant. All data analyses were performed using SPSS (version 25.0; IBM Corp) on an intention-to-treat basis.

### Ethical Considerations

This study was approved by the Medical Ethics Committee of Peking Union Medical College Hospital, Chinese Academy of Medical Sciences (ZS-3411).

## Results

### Participant Flow and Baseline Characteristics

**Figure 3.**
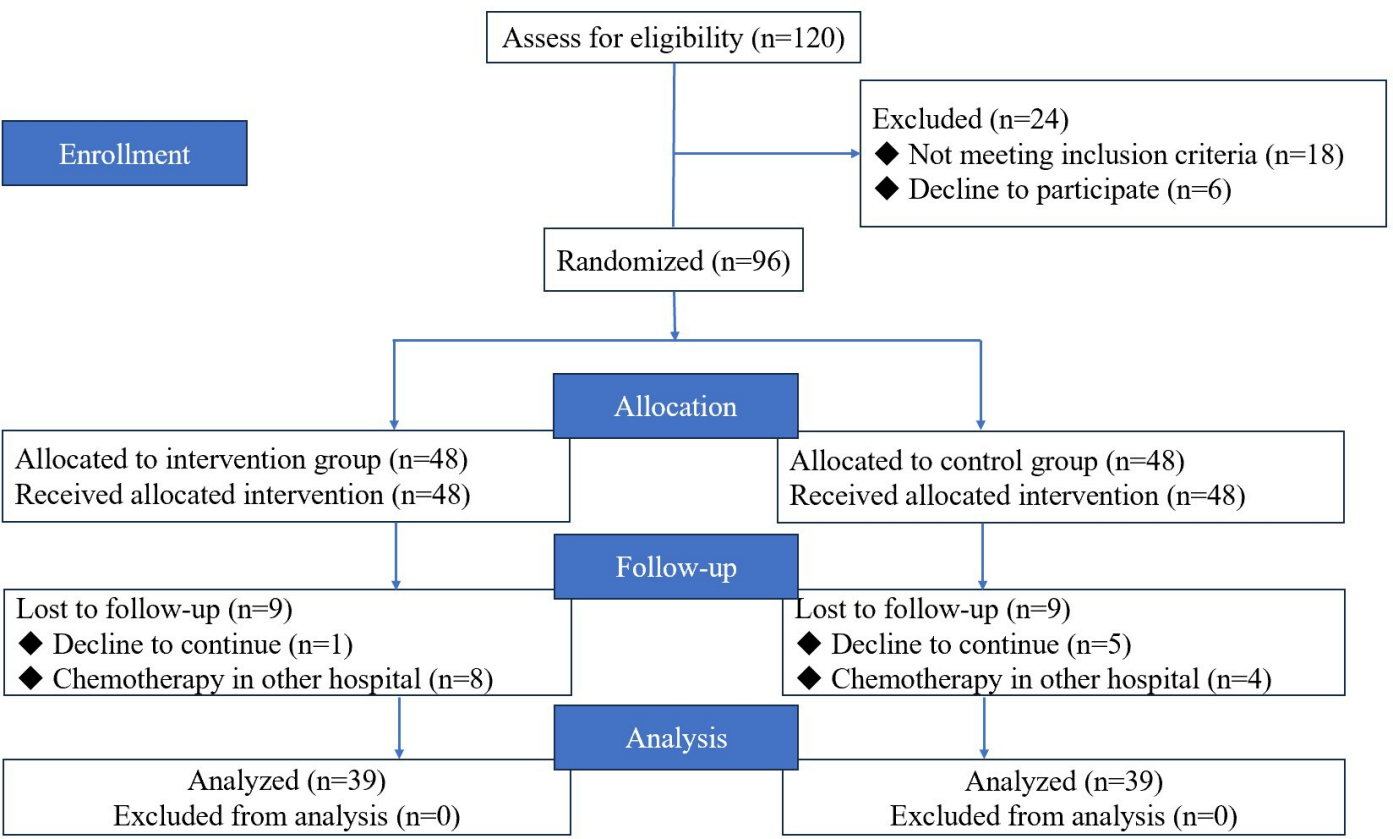
Flowchart of recruitment.

A total of 120 patients were notified of the study before they were admitted to the hospital for the first chemotherapy treatment, of which 18 patients did not fulfill the inclusion criteria and 6 patients refused to participate in the study. Therefore, 96 patients were involved in the random allocation, that is, 48 patients each in the intervention and control groups. During the follow-up, there was sample dropout in both groups: 1 (1/48, 2.1%) patient and 5 (5/48, 10.4%) patients in both groups who refused to continue participating in the study, in addition to 8 (8/48, 16.67%) patients and 4 (4/48, 8.3%) patients in both groups who went to other hospitals for chemotherapy. The flow diagram is shown in Figure 3. There were no statistically significant differences in demographic and clinical characteristics between the intervention and control groups, as shown in Table 2.

**Table 2.** Demographic and clinical characteristics.

Variables	Intervention group (n=39)	Control group (n=39)	Chi-square (*df*)/*Z*	*P* value
Age (years), median (IQR)	55.00 (49.00-60.00)	56.00 (48.00-65.00)	−0.50^a^	.62
BMI (kg/m^2^), median (IQR)	22.48 (20.45-24.40)	22.14 (19.78-24.50)	−0.71^a^	.48
**Marital status, n (%)**			0.000 (38)^b^	>.99
Married	37 (94.9)	37 (94.9)	N/A^c^	N/A
Unmarried, divorced, or widowed	2 (5.1)	2 (5.1)	N/A	N/A
**Education level, n (%)**			0.56 (38)^b^	.76
Junior high school and below	13 (33.3)	10 (25.6)	N/A	N/A
High school	11 (28.2)	12 (30.8)	N/A	N/A
College degree and above	15 (38.5)	17 (43.6)	N/A	N/A
**Number of children, n (%)**			0.47 (38)^b^	>.99
0	1 (2.6)	2 (5.1)	N/A	N/A
1	25 (64.1)	25 (64.1)	N/A	N/A
≥2	13 (33.3)	12 (30.8)	N/A	N/A
**Family monthly income per capita (¥^d^), n (%)**			1.39 (38)^b^	.71
≤2000	7 (18.0)	7 (18.0)	N/A	N/A
2001-4000	9 (23.1)	9 (23.1)	N/A	N/A
4001-6000	10 (25.6)	14 (35.8)	N/A	N/A
>6000	13 (33.3)	9 (23.1)	N/A	N/A
**Professional status, n (%)**			0.83 (38)^b^	.36
Active employee	19 (48.7)	24 (61.54)	N/A	N/A
Inactive employee	20 (51.3)	15 (38.46)	N/A	N/A
**Type of medical insurance, n (%)**			1.46 (38)^b^	.74
Free medical care	5 (12.8)	2 (5.1)	N/A	N/A
Beijing Medical Insurance	8 (20.5)	8 (20.5)	N/A	N/A
Remote medical insurance	20 (51.3)	22 (56.4)	N/A	N/A
New rural cooperative medical insurance	6 (15.4)	7 (18.0)	N/A	N/A
**Residence, n (%)**			0.11 (38)^b^	.75
Town	33 (84.6)	34 (87.2)	N/A	N/A
Rural	6 (15.4)	5 (12.8)	N/A	N/A
**Stage, n (%)**			0.87 (38)^b^	.87
I	5 (12.8)	5 (12.8)	N/A	N/A
II	15 (38.5)	17 (43.6)	N/A	N/A
III	17 (43.6)	13 (33.3)	N/A	N/A
IV	2 (5.1)	4 (10.3)	N/A	N/A
**Surgical procedure, n (%)**			0.05 (38)^b^	.82
Laparoscopy	19 (48.7)	18 (46.1)	N/A	N/A
Laparotomy	20 (51.3)	21 (53.9)	N/A	N/A

a *Z* value.

b Chi-square value.

c N/A: not applicable.

d A currency exchange rate of ¥1=US $0.14 is applicable.

### Nutritional Status—PG-SGA Score

**Figure 4.**
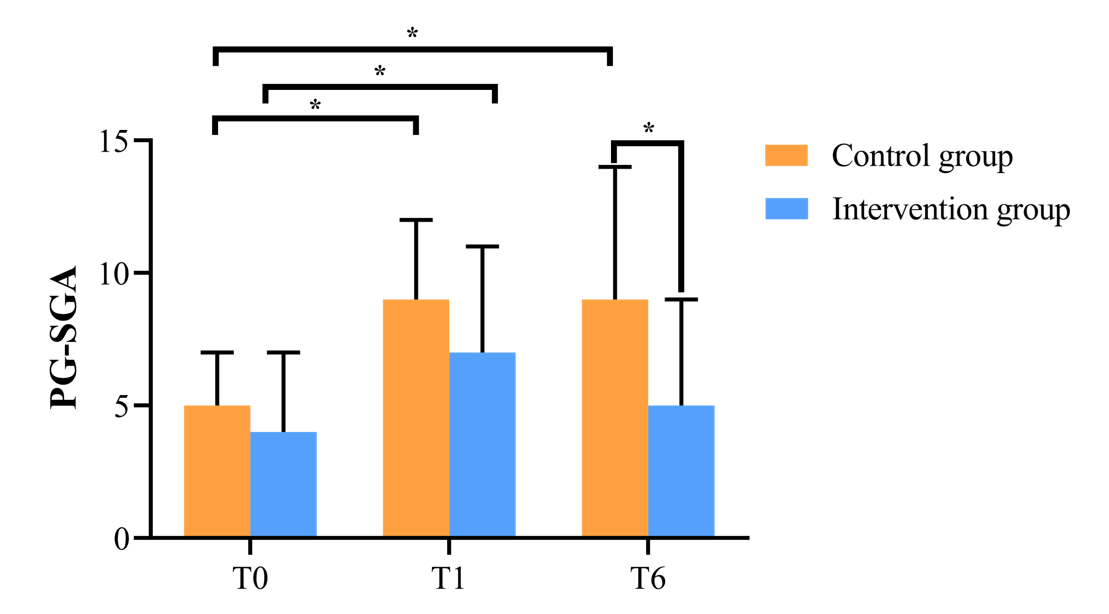
Changes in PG-SGA scores at T0, T1, and T6 between intervention and control groups. T0=prechemotherapy; T1=after first chemotherapy; and T6=after sixth chemotherapy. *There was a significant difference between the intervention and control groups (*P*<.05). PG-SGA: Patient-Generated Subjective Global Assessment.

Figure 4 demonstrates the PG-SGA scores of the intervention and control groups at T0, T1, and T6. Among the 3 time points, there was a statistically significant difference in PG-SGA scores between the 2 groups only at T6. By longitudinal comparison, we found that the difference of PG-SGA scores was statistically significant in the intervention group between T0 and T1 (*P*<.05), as well as in the control group, with scores lower than the T0 period. However, comparing T0 and T6, only T6 in the control group had a lower score than T0 (*P*<.05). The mixed linear model revealed that the group-based effect on PG-SGA scores was significant (*F*_47_=4.763; *P*=.03), whereas the time-based effect on PG-SGA scores was not (*F*_47_=0.377; *P*=.54). The group-time interaction effect on PG-SGA scores was statistically significant (*F*_46_=6.368; *P*=.01) (Table 3).

**Table 3.** Changes within groups and comparisons between groups regarding the Patient-Generated Subjective Global Assessment (PG-SGA) scores: linear mixed model analysis^a^.

Groups	T0 (n=39)	T1 (n=39)		T6 (n=39)	
	Score, median (IQR)	Score, median (IQR)	Change from T0, median difference (95% CI)	Score, median (IQR)	Change from T0, median difference (95% CI)
Intervention	4.00 (3.00 to 7.00)	7.00 (4.00 to 11.00)	−3.00 (−4.00 to 0.00)	5.00 (3.00 to 9.00)	−1.00 (−2.00 to 1.00)
Control	5.00 (2.00 to 7.00)	9.00 (2.00 to 12.00)	−4.00 (−5.00 to 0.00)	9.00 (4.00 to 14.00)	−4.00 (−6.00 to −2.00)
Change between groups,median difference (95% CI)	1.00 (−1.00 to 2.00)	2.00 (−2.00 to 2.00)	N/A^b^	4.00 (1.00 to 5.00)	N/A

a Linear mixed model was used for the analysis of changes within group and comparisons between groups of the PG-SGA scores, with T0 measurement of the PG-SGA scores as covariate; group, time, and group × time interaction as fixed effects; and patient as random effect. PG-SGA: (group) *F*_47_=4.763, *P*=.03; (time) *F*_47_=0.377, *P*=.54; (group × time interaction) *F*_46_=6.368, *P*=.01.

b N/A: not applicable.

### Compound Immune-Nutritional and Compound Immune-Inflammatory Indicators in Blood

**Figure 5.**
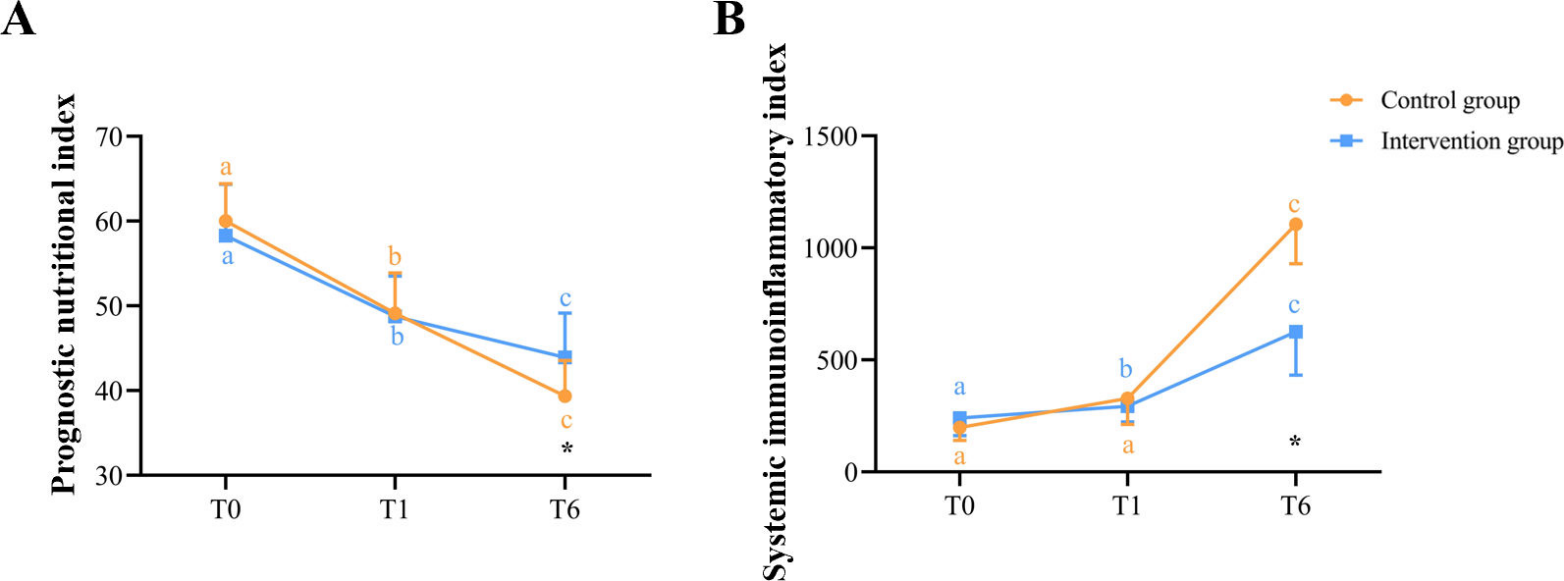
Changes in compound immune-nutritional and compound immune-inflammatory indicators in blood at T0, T1, and T6 between intervention and control groups. The line graphs show the differences and changes in prognostic nutritional index (**A**) and systemic immunoinflammatory index (**B**) between the intervention and control groups at T0, T1, and T6. *There was a significant difference between the intervention and control groups (*P*<.05). With regard to “a,” “b,” and “c,” different letters at the 2 time points indicate that there is a significant difference in the blood indices of the intervention or control group at the 2 time points (*P*<.05).

Among the 3 time points, there was a statistically significant difference in the PNI and SII between the 2 groups only at T6. By longitudinal comparison, there was a statistically significant difference in the comparison between all 3 time points for the PNI (Figure 5A) and SII (Figure 5B) in the intervention group, as well as in the control group (*P*<.05) (Figure 5). The mixed linear model revealed that the group-based effect on nutrition-inflammation composite indices was significant (*F*_47_=7.653, *P*=.006; *F*_47_=13.309, *P*<.001), as well as the time-based effect on nutrition-inflammation composite indices (*F*_47_=92.304, *P*<.001; *F*_47_=110.675, *P*<.001). The group-time interaction effect on nutrition-inflammation composite indices was statistically significant (*F*_46_=10.379, *P*=.002; *F*_46_=5.289, *P*=.02) (Table 4).

**Table 4.** Changes within groups and comparisons between groups regarding the nutrition-inflammation composite indices: linear mixed model analysis^a^.

Groups	T0 (n=39)	T1 (n=39)		T6 (n=39)	
	Score, median (IQR)	Score, median (IQR)	Change from T0,median difference (95% CI)	Score, median (IQR)	Change from T0,median difference (95% CI)
**PNI** ^b^					
Intervention	57.95 (53.50 to 61.00)	48.90 (44.55 to 52.80)	9.05 (6.550 to 11.50)	44.10 (41.20 to 47.35)	13.85 (11.45 to 16.60)
Control	60.40 (56.70 to 63.05)	48.5 (46.2 to 51.25)	11.9 (9.20 to 13.30)	39.85 (36.80 to 42.65)	20.55 (18.80 to 22.55)
Change between groups, median difference (95% CI)	−2.45 (−0.15 to 4.80)	0.4 (−2.20 to 2.35)	N/A^c^	−4.25 (−6.70 to −2.25)	N/A
**SII** ^d^					
Intervention	241.07 (161.70 to 306.38)	293.6834 (223.17 to 382.31)	−52.612 (−113.46 to −16.37)	625.37 (432.02 to 1273.21)	−384.30 (−679.19 to −280.35)
Control	197.6443 (140.86 to 327.68)	328.31 (212.17 to 552.86)	−130.66 (−206.08 to −58.14)	1106.15 (929.42 to 1672.49)	−908.51 (−1026.72 to −785.50)
Change between groups, median difference (95% CI)	−43.4271 (−72.70 to 27.57)	34.6246 (−31.67 to 127.02)	N/A	480.7798 (213.29 to 624.26)	N/A

a Linear mixed model was used for the analysis of changes within group and comparisons between groups of the nutrition-inflammation composite indices, with T0 measurement of the nutrition-inflammation composite indices as covariate; group, time, and group × time interaction as fixed effects; and patient as random effect.

b PNI (prognostic nutritional index): (group) *F*_47_=7.653, *P*=.006; (time) *F*_47_=92.304, *P<*.001; (group × time interaction) *F*_46_=10.379, *P*=.002.

c N/A: not applicable.

d SII (systemic immunoinflammatory index): (group) *F*_47_=13.309, *P*<.001; (time) *F*_47_=110.675, *P*<.001; (group × time interaction) *F*_46_=5.289, *P*=.02.

### Nutrition-Related and Inflammation-Related Blood Indices

**Figure 6.**
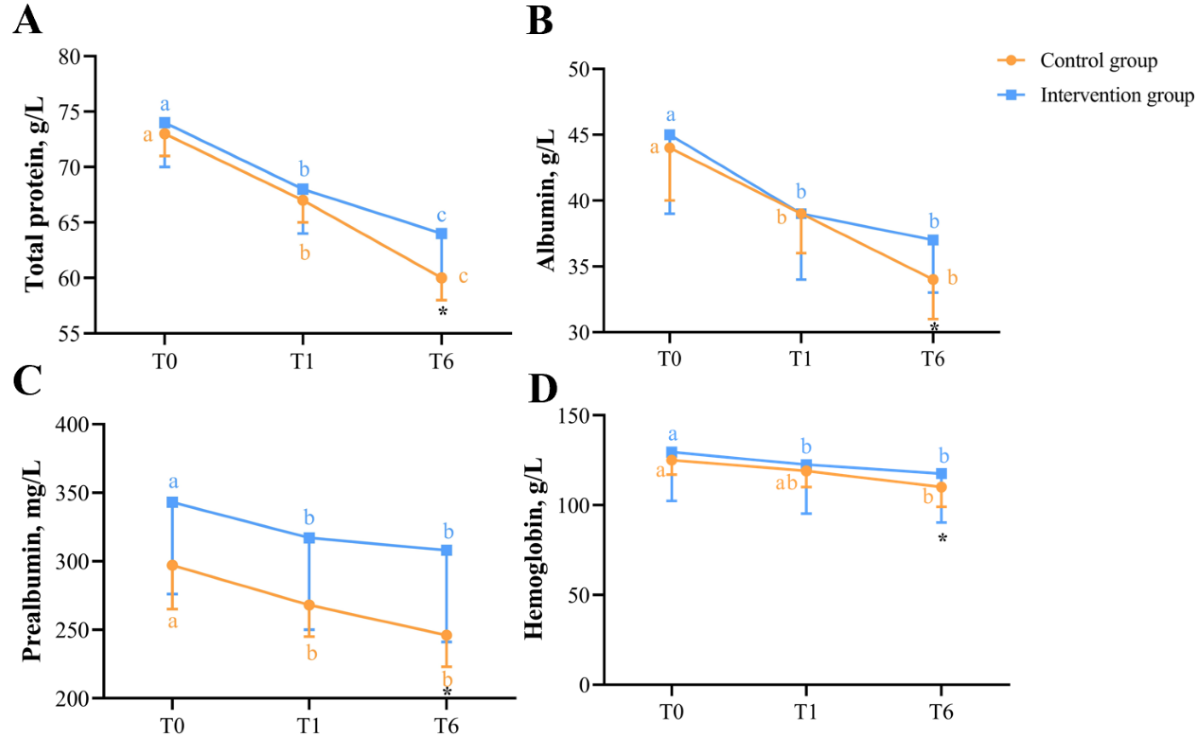
Changes in nutritional indicators in blood at T0, T1, and T6 between intervention and control groups. The line graphs show the differences and changes in total protein (**A**), albumin (**B**), prealbumin (**C**), and hemoglobin (**D**) between the intervention and control groups at T0, T1, and T6. *There was a significant difference between the intervention and control groups (*P*<.05). With regard to “a, “b,” and “c,” different letters at the 2 time points indicate that there is a significant difference in the blood indices of the intervention or control group at the 2 time points, whereas the same letter indicates that there was no significant difference (*P>*.05).

**Figure 7.**
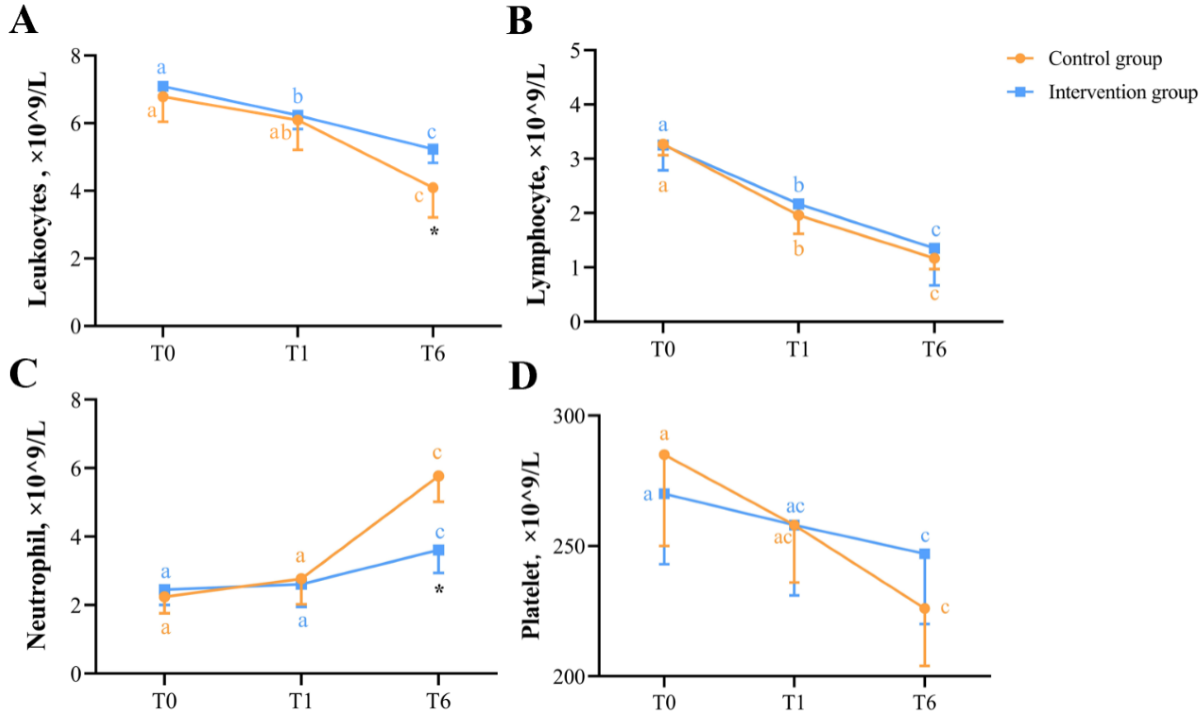
Changes in inflammatory parameters in blood at T0, T1, and T6 between intervention and control groups. The line graphs show the differences and changes in white blood cells (**A**), lymphocytes (**B**), neutrophils (**C**), and platelets (**D**) between the intervention and control groups at T0, T1, and T6. *There was a significant difference between the intervention and control groups (*P*<.05). With regard to “a,” “b,” and “c,” different letters at the 2 time points indicate that there is a significant difference in the blood indices of the intervention or control group at the 2 time points (*P*<.05).

Changes in blood indices related to nutrition and inflammation at T0, T1, and T6 are shown in Figures 6 and 7, respectively. Figure 6 showed the differences and changes in total protein (Figure 6A), albumin (Figure 6B), prealbumin (Figure 6C), and hemoglobin (Figure 6D) between the intervention and control groups at T0, T1, and T6. Figure 7 showed the differences and changes in white blood cells (Figure 7A), lymphocytes (Figure 7B), neutrophils (Figure 7C), and platelets (Figure 7D) between the intervention and control groups at T0, T1, and T6. The mixed linear model displayed that the group-based effect on total protein (*F*_47_=19.712; *P<*.001), prealbumin (*F*_47_=10.029; *P*=.002), hemoglobin (*F*_47_=6.225; *P*=.01), lymphocytes (*F*_47_=3.921; *P*=.05), and neutrophils (*F*_47_=72.058; *P*<.001) was significant. The time-based effect on total protein (*F*_47_=75.642; *P*<.001), albumin (*F*_47_=24.496; *P*<.001), prealbumin (*F*_47_=5.054; *P*=.03), hemoglobin (*F*_47_=27.744; *P*<.001), leukocytes (*F*_47_=29.950; *P*<.001), lymphocytes (*F*_47_=76.220; *P<*.001), neutrophils (*F*_47_=182.218; *P<*.001), and platelets (*F*_47_=9.350; *P*=.003) was statistically significant. The group-time interaction effect was statistically significant on total protein (*F*_46_=5.626; *P*=.02), albumin (*F*_46_=7.346; *P*=.007), hemoglobin (*F*_46_=6.225; *P*=.01), leukocytes (*F*_46_=3.328; *P*=.07), and neutrophils (*F*_46_=45.555; *P*<.001). Detailed information is given in Table S2 in Multimedia Appendix 2.

## Discussion

### Principal Findings

Owing to the difficulty of uniformly managing patients with OC undergoing chemotherapy during the chemotherapy intervals, remote and easily accessible alternatives are needed to improve the nutritional status of patients with OC undergoing chemotherapy. This study of patients with OC undergoing chemotherapy analyzed the effect of a full-course nutritional management program based on the WeChat platform on nutritional status. The results obtained showed that the interventions led to significant improvements in nutritional status in terms of reducing PG-SGA scores, increasing nutrition-related blood indices, and decreasing inflammation-related blood indices.

### Comparison With Previous Work

Patients in both the intervention and control groups showed significant increases in the PG-SGA scores after the first chemotherapy compared with baseline, demonstrating that the nutritional status was markedly impaired in patients undergoing chemotherapy. This finding was consistent with those of other related studies [35-37]. The 2 main reasons are analyzed as follows. On the one hand, gastrointestinal symptoms such as nausea and vomiting caused by the paclitaxel-combined carboplatin chemotherapy regimen are the most pronounced and severe after the first chemotherapy session [38], which limits the patient’s nutrient intake and absorption, resulting in a loss of body weight and muscle mass, and an elevated PG-SGA score. On the other hand, patients may also develop a cluster of peripheral neurological symptoms after the first chemotherapy session, affecting their motor function [39].

There was no significant difference in PG-SGA scores between the 2 groups after the first chemotherapy treatment, suggesting that the short-term benefits of nutritional intervention strategies have not yet been demonstrated. In addition to the adverse effects of chemotherapy, some patients may experience the following symptoms after their first chemotherapy treatment: anxiety, depression, pain, sexual dysfunction, fatigue, personality changes, and other symptoms [40]. Their normal adaptive mechanisms are challenged and their future planning is compromised, leading to a reduction in their disease self-efficacy [41].

At the sixth chemotherapy follow-up visits, the PG-SGA scores of the intervention group were not significantly different from those at baseline, indicating that the program helped patients gradually return to their prechemotherapy nutritional status during the sixth course of chemotherapy. Meanwhile, the PG-SGA scores of the intervention group remained significantly lower than those of the control group at sixth chemotherapy. This result was consistent with the study by Wang et al [42]. Similarly, we found significant differences between the 2 groups after the sixth session of chemotherapy for both indicators, the PNI and SII. After the sixth chemotherapy session, the intervention group had a higher PNI level and a lower SII level than the control group, which suggested that the nutritional intervention program during chemotherapy may further improve the long-term nutritional prognosis of patients after chemotherapy. The WeChat platform was divided into 3 main interventions: WeChat applet, WeChat group chat, and private message. First of all, the health education content of the health education module in the WeChat applet was a comprehensive, detailed, and authoritative holistic nutritional intervention program during chemotherapy for patients to review and learn through literature review and expert correspondence, so as to enhance their nutritional literacy. The section was presented in the form of text combined with pictures to easily stimulate patients’ interest in reading. The knowledge subscription section was for patients to subscribe to the content according to their own interests and symptoms after chemotherapy, so that patients could read and learn repeatedly anytime and anywhere. The questionnaire test was to regularly check the learning effect of patients, so as to provide targeted intervention. The joint intervention of the 3 sections improved patients’ nutritional literacy during chemotherapy, thus realizing the improvement of nutritional status. Compared with Keum et al [43], who intervened in patients’ nutritional status through a smartphone app, the WeChat applet in our study was more convenient and economical as it did not require downloads. Second, the WeChat group chat broke through the limitations of time and space to provide a platform for patients to communicate anytime, anywhere [44]. This platform enabled patients to share nutritional experiences, released negative emotions, and gained encouragement from each other [45]. In addition, nutritional team members would correct incorrect nutritional concepts and knowledge in the group chat in a timely manner. Relevant discussions in the group chat could be retained in the WeChat group for a long period of time, which facilitated repeated learning. Peer support from health care professionals and patients in the WeChat group chat also enhanced patients’ trust and reliance on the nutrition intervention program [46]. The private massage provided a platform for patients to consult and took timely and effective countermeasures when they encountered nutritional problems [47].

### Limitations and Strengths

There are some limitations in our study. First, to participate in the WeChat-based nutrition intervention program, patients must have mobile devices with web access, which suggested that the patients were younger or have supportive family members. Second, the sample size was small. A barrier to recruiting more participants is that most patients choose to go home for follow-up chemotherapy due to their financial situation, and it is difficult to access the nutritional status of patients. A larger sample size could enhance the knowledge gleaned about the efficacy of the intervention. In addition, our study captured only patients’ self-reported objective physical outcomes such as weight and specific types of chemotherapy-related symptoms and did not assess patients’ psychological perceptions of their nutritional status. In-depth qualitative interviews may be able to compensate for the bias in outcomes. Finally, the study was conducted in a public general tertiary care hospital, so it is necessary to test whether the good results observed can be generalized to other health care institutions.

This study also has some strengths. First, the study site was in a large general tertiary hospital that received patients from all over the country, so the study samples were representative of patients with OC undergoing chemotherapy with malnutrition conditions occurring in different regions of China. In addition, the nutritional assessment tool in the study has been internationally validated, thus allowing for replication and comparison with other studies. Another advantage was that the trial was conducted through a WeChat applet, which was easy to use and did not require downloading an additional application. In addition, the study included only those patients undergoing chemotherapy for OC, so the patient sample was more homogeneous than studies that included patients undergoing chemotherapy for gynecologic oncology.

### Conclusions

In this study, a normative WeChat-based nutritional management program lasting for 6 chemotherapy intervals significantly improved nutritional status in patients with OC undergoing chemotherapy compared with conventional controls. Improving personalized nutritional intervention strategies in standard nutritional management protocols for patients with OC and introducing the WeChat platform as a mobile intervention model can enhance patients’ nutritional status during chemotherapy and improve their quality of life.

## Supplementary material

10.2196/56475 Multimedia Appendix 1 Informed consent form for this study.

10.2196/56475 Multimedia Appendix 2 Nutritional program and nutrition-related and inflammation-related blood indices.

10.2196/56475 Checklist 1 CONSORT-eHEALTH checklist (V 1.6.1).

## Abbreviations

OC: ovarian cancer
PG-SGA: Patient-Generated Subjective Global Assessment
PNI: prognostic nutritional index
SII: systemic immunoinflammatory index
