# Supplementary material for: Efficacy of a WeChat-Based, Multidisciplinary, Full-Course Nutritional Management Program on the Nutritional Status of Patients With Ovarian Cancer Undergoing Chemotherapy: Randomized Controlled Trial
Source: JMIR Mhealth Uhealth. 2024 Nov 4;12:e56475. doi: 10.2196/56475 (PMC11554286; doi:10.2196/56475)
Supplement: Multimedia Appendix 1 [file mhealth-v12-e56475-s001.doc]

**受试者知情同意书**

**研究项目名称**：基于移动医疗的妇科恶性肿瘤化疗患者营养管理模式构建及应用效果评价

**研究负责人**：田小娟

**联系电话**：69155735

**研究单位**：中国医学科学院北京协和医院

1．研究背景、目的：

化疗和肿瘤往往会导致各种程度的营养不良。本研究旨在应用微信小程序对化疗患者进行营养管理，早期筛查出患者是否存在营养不良的危险因素。

2．研究内容、方法及程序：

入组后，由工作人员指导关注微信小程序，并和工作人员添加微信好友，首次填写一般资料调查表及患者主观整体营养状况评估量表，然后于化疗期间于小程序在线填写化疗副反应、实验室检验指标和患者主观整体营养状况评估量表。

3．参加研究的可能风险（或不适、不便）和受益（个人或社会群体受益）：

本研究不会对患者身体造成额外的伤害，通过科学的管理模式对患者进行营养管理，可以尽早发现患者是否存在营养不良的危险因素。

4．有关内容的咨询：您有权就有关研究内容进行咨询，咨询电话： 69155735；且您有权就有关您的权利或相关风险等问题进行咨询，咨询电话（伦理审查委员会办公室电话）：69156874。

5．退出研究的权利：您参加此项研究是完全自愿的。无需任何原因，您不愿意参加或不愿继续参加此研究，并不会对您的权益有任何影响。此外，您有权在任何时间退出此研究。

6．研究的赔偿：本研究未增加治疗以外的风险，所以不涉及赔偿事宜。

7．保密制度：您参加此研究所获得的医疗信息将得到保密。研究结果在学术刊物上发表时也不会泄露任何可识别您个人身份的信息。北京协和医院将保存您在这项研究中的全部记录以及有关的医院和办公室记录，未经授权任何人不得获取这些信息。

8．本知情同意书一式两份，受试者和研究者各一份，双方签字后有效。

**受试者的知情同意：**

我已详细阅读并充分了解以上内容，并对以上内容，特别是我参与此研究的权利、风险和受益进行了认真考虑。我自愿参加这项研究，愿意与研究人员合作。同时声明我可以在任何时候因任何原因退出此研究，而不会丧失任何合法权利。

受试者姓名(正楷)： 受试者签字： 日期： 年 月 日

研究者姓名(正楷)： 研究者签字： 日期： 年 月 日

**Subject's informed consent**

Title of Research Project: Construction of a Nutritional Management Model for Chemotherapy Patients with Gynaecological Malignant Tumours Based on Mobile Healthcare and Evaluation of the Application Effects

Research Leader: Tian Xiaojuan

Tel: 69155735

Research unit: Peking Union Medical College Hospital, Chinese Academy of Medical Sciences, Beijing, China

1. Research background, purpose:

Chemotherapy and tumours often lead to various degrees of malnutrition. The aim of this study is to apply the WeChat applet for nutritional management of chemotherapy patients, and to screen out the presence of malnutrition risk factors in patients at an early stage.

2. study content, methods and procedures:

After enrollment, the staff instructed to pay attention to the WeChat applet and add WeChat friends with the staff, fill in the general information questionnaire and the patient's subjective overall nutritional status assessment scale for the first time, and then fill in the chemotherapeutic side-effects, laboratory test indexes, and the patient's subjective overall nutritional status assessment scale online at the applet during the chemotherapy period.

3. Possible risks (or discomfort, inconvenience) and benefits (individual or social group benefits) of participating in the study:

This study will not cause any additional harm to the patient's body, and the nutritional management of the patient through a scientific management model will allow early detection of the presence of risk factors for malnutrition.

4. Consultation about the content: You have the right to consult about the content of the study at 69155735, and you have the right to consult about your rights or the related risks at 69156874 (Office of the Ethics Review Committee).

5. Right to withdraw from the study: Your participation in this study is completely voluntary. There is no reason why you do not want to participate or continue to participate in this study, and this will not affect your rights in any way. In addition, you have the right to withdraw from this study at any time.

6. Compensation for the study: This study does not involve compensation because it does not increase risk beyond the treatment.

7. Confidentiality: Medical information obtained from your participation in this study will be kept confidential. The results of the study will be published in academic journals without disclosing any personally identifiable information about you. Peking Union Medical College Hospital will keep all records of your participation in this study, as well as relevant hospital and office records, and no one will be allowed to access this information without authorisation.

8. Two copies of this informed consent form, one for the subject and one for the investigator, will be validly signed by both parties.

Subject's Informed Consent:

I have read and fully understand the above in detail and have given careful consideration to the above, particularly the rights, risks and benefits of my participation in this study. I volunteer to participate in this study and am willing to co-operate with the researchers. It is also declared that I may withdraw from this study at any time for any reason without loss of any legal rights.

Subject's name (in block letters): Subject's signature: Date: Month of year

Researcher's name (in block letters): Researcher's signature: Date: Month/Year

Translated with DeepL.com (free version)
