# Supplementary material for: Efficacy of a WeChat-Based, Multidisciplinary, Full-Course Nutritional Management Program on the Nutritional Status of Patients With Ovarian Cancer Undergoing Chemotherapy: Randomized Controlled Trial
Source: JMIR Mhealth Uhealth. 2024 Nov 4;12:e56475. doi: 10.2196/56475 (PMC11554286; doi:10.2196/56475)
Supplement: Multimedia Appendix 2 [file mhealth-v12-e56475-s002.zip › Supplementary Table 2.docx]

**Supplementary Table 2：Changes within groups and comparisons between groups of the nutrition-related and inflammation-related blood indices: linear mixed model analysis.**

| Groups | T0 (n=39) |  | T1 (n=39) | |  | T6 (n=39) | |
| --- | --- | --- | --- | --- | --- | --- | --- |
|  | Score |  | Score | Change from baseline (95% CI) |  | Score | Change from baseline (95% CI) |
| **Nutrition-related blood indices** |  |  |  |  |  |  |  |
| Total protein (g/L) |  |  |  |  |  |  |  |
| Intervention | 74.0 (70, 76) |  | 68 (64, 70) | -6 (4, 8) |  | 64 (60, 66) | -10 (8, 12) |
| Control | 73 (71, 74) |  | 67 (65, 69) | -6 (5, 8) |  | 60 (58, 62) | -13 (12, 15) |
| Change between groups (95% CI) | 1 (-3, 1) |  | 1 (-3.000, 0.000) |  |  | 4 (-6, -3) |  |
| Albumin (g/L) |  |  |  |  |  |  |  |
| Intervention | 45 (39, 47) |  | 39 (34, 41) | -6 (4, 8) |  | 37 (33, 39) | -8 (5, 9) |
| Control | 44 (40, 47) |  | 39 (36, 41) | -5 (4, 7) |  | 34 (31, 36) | -10 (9, 12) |
| Change between groups (95% CI) | 1 (-2, 2) |  | 0 (-1, 2) |  |  | 3 (-5, -1) |  |
| Prealbumin (g/L) |  |  |  |  |  |  |  |
| Intervention | 343 (276 364) |  | 317 (250, 338) | -26 (12, 40) |  | 308 (241, 329) | -35 (21, 49) |
| Control | 297 (265, 345) |  | 268 (245, 317) | -29 (9, 40) |  | 246 (223, 295) | -51 (31, 62) |
| Change between groups (95% CI) | -46 (-32, 0) |  | -49 (-33, 2) |  |  | -62 (-46, -11) |  |
| Hemoglobin (g/L) |  |  |  |  |  |  |  |
| Intervention | 127 (117, 132) |  | 120 (110, 125) | -7 (2,12) |  | 115 (105, 120) | -12 (7, 17) |
| Control | 123 (117, 129) |  | 121 (113, 125) | -2 (-2, 7) |  | 107 (99, 111) | -16 (12, 21) |
| Change between groups (95% CI) | 4 (-7, 2) |  | -1 (-3, 5) |  |  | 8 (-12, -3) |  |
| **Inflammation-related blood indices** |  |  |  |  |  |  |  |
| Leukocytes |  |  |  |  |  |  |  |
| Intervention | 7.09 (6.81, 8.05) |  | 6.23 (5.82, 6.84 | -0.86 (0.58, 1.36) |  | 5.23 (4.82, 5.84) | -1.86 (1.580, 2.360) |
| Control | 6.78 (6.04, 8.64) |  | 6.09 (5.21, 7.46) | -0.69 (-0.09, 1.57) |  | 4.09 (3.21, 5.46) | -2.69 (1.910, 3.570) |
| Change between groups (95% CI) | 0.31 (-0.99, 0.25) |  | 0.14 (-0.70, 0.63) |  |  | 1.14 (-1.7, -0.37) |  |
| Lymphocytes |  |  |  |  |  |  |  |
| Intervention | 3.25 (2.79, 4.21) |  | 2.17 (1.97, 2.33) | -1.08 (0.91, 1.47) |  | 1.35 (0.67, 2.13) | -1.9 (1.61, 2.37) |
| Control | 3.27 (3.07, 3.43) |  | 1.96 (1.62, 2.45) | -1.31 (1.08, 1.50) |  | 1.17 (0.97, 1.33) | -2.1 (1.98, 2.22) |
| Change between groups (95% CI) | -0.02 (-0.37, 0.19) |  | 0.21 (-0.40, 0.02) |  |  | 0.18 (-0.590, 0.120) |  |
| Neutrophils |  |  |  |  |  |  |  |
| Intervention | 2.45 (2.00, 3.33) |  | 2.61 (1.94, 3.10) | 0.16 (-0.47,0.36) |  | 2.45 (2.00, 3.33) | 0 (-1.47, -0.64) |
| Control | 2.24 (1.76, 3.36) |  | 2.77 (2.02, 3.45) | 0.53 (-0.76, 0.21) |  | 5.77 (5.02, 6.45) | 3.53 (-3.76, -2.79) |
| Change between groups (95% CI) | 0.21 (-0.52, 0.43) |  | -0.16 (-0.21, 0.64) |  |  | -3.32 (1.79, 2.64) |  |
| Platelets |  |  |  |  |  |  |  |
| Intervention | 270 (243, 289) |  | 258 (231, 277) | -12 (-4, 28) |  | 247 (220, 266) | -23 (7, 39) |
| Control | 285 (250, 329) |  | 258 (236, 302) | -27 (-4, 46) |  | 226 (204, 270) | -59 (28, 78) |
| Change between groups (95% CI) | -15 (-5, 38) |  | 0 (-16, 26) |  |  | 21 (-37, 5) |  |

95% CI: 95% confidence interval.

Linear mixed model was used for the analysis of changes within group and comparisons between groups of the PG-SGA scores, with baseline measurement of the PG-SGA scores as covariate; group, time, and group × time interaction as fixed effects; and patient as random effect.

Total protein: (group) F = 19.712, *P*＜0.001; (time) F = 75.642, *P*＜0.001; (group × time interaction) F = 5.626, *P* = 0.019.

Albumin: (group) F = 3.265, *P* = 0.073; (time) F = 24.496, *P*＜0.001; (group × time interaction) F = 7.346, *P* = 0.007.

Prealbumin: (group) F = 10.029, *P* = 0.002; (time) F = 5.054, *P*=0.026; (group × time interaction) F = 0.889, *P* = 0.347.

Hemoglobin: (group) F = 0.976, *P* = 0.325; (time) F = 27.744, *P*＜0.001; (group × time interaction) F = 6.225, *P* = 0.014.

Leukocytes: (group) F = 1.239, *P* = 0.267; (time) F = 29.950, *P*＜0.001; (group × time interaction) F = 3.328, *P* = 0.07.

Lymphocytes: (group) F = 3.921, *P* = 0.050; (time) F = 76.220, *P*＜0.001; (group × time interaction) F = 2.127, *P* = 0.147.

Neutrophils: (group) F = 72.058, *P*＜0.001; (time) F = 182.218, *P*＜0.001; (group × time interaction) F = 45.555, *P*＜0.001.

Platelets: (group) F = 1.031, *P* = 0.311; (time) F = 9.350, *P*=0.003; (group × time interaction) F = 2.230, *P* = 0.137.
