# Supplementary material for: Efficacy of a WeChat-Based, Multidisciplinary, Full-Course Nutritional Management Program on the Nutritional Status of Patients With Ovarian Cancer Undergoing Chemotherapy: Randomized Controlled Trial
Source: JMIR Mhealth Uhealth. 2024 Nov 4;12:e56475. doi: 10.2196/56475 (PMC11554286; doi:10.2196/56475)
Supplement: Multimedia Appendix 2 [file mhealth-v12-e56475-s002.zip › Supplementary Table1 (Revised Version).docx]

**Supplementary Table 1.** **A multidisciplinary full-course nutritional management program for ovarian cancer patients undergoing chemotherapy**

| **Indicators** | **Significance score (x ̅±s)** | **Coefficient of variation** |
| --- | --- | --- |
| **1 Nutritional screening and assessment** | 5.0 | 0 |
| **1.1 Personnel** It is recommended that a multidisciplinary team consisting of clinicians, care managers, dietetic nurses, dietitians, and rehabilitation therapists be formed and that team members be responsible for screening and assessing patients for nutritional risk [1-8] | 4.75±0.45 | 0.095 |
| **1.2 Nutritional screening** | 5.0 | 0 |
| **1.2.1 Nutritional risk** screening is performed using the NRS 2002 and is completed within 24h of admission [1, 7, 8] | 5.0 | 0 |
| 1.2.2 Patients with NRS 2002 score <3 who are not at nutritional risk are screened once a week [2, 7, 8] | 4.75±0.45 | 0.095 |
| 1.2.3 Patients with NRS 2002 score ≥3 at nutritional risk undergo further nutritional assessment [2, 3, 6-8] | 4.83±0.39 | 0.081 |
| 1.3 Nutritional assessment comprehensive nutritional assessment using PG-SGA [3, 6-8] | 5.0 | 0 |
| **2 Nutrient Requirements** | 5.0 | 0 |
| **2.1 Energy** Patients' energy requirements were individually assessed using The Mifflin-St Jeor formula [8] | 5.0 | 0 |
| **2.2 Carbohydrates and fats** Refer to healthy population, but increase fat and decrease carbohydrate intake in patients with insulin resistance and weight loss [2, 8] | 3.92±0.67 | 0.171 |
| **2.3 Protein** Recommended intake >1g/(kg-d), with 1.5-2.0g/(kg-d) recommended for severe depletion; however, protein supplements should not exceed 1.0 and 1.2g/(kg-d) for patients with acute or chronic renal failure, respectively [1, 2, 7, 8] | 5.0 | 0 |
| **2.4 Micronutrients** Suggested intake is the recommended amount for normal daily intake; routine use of micronutrient supplements is not recommended in the absence of deficiency [2, 7, 9] | 4.67±0.49 | 0.105 |
| **3 Nutritional interventions** | 5.0 | 0 |
| **3.1 Timing** The presence of the following conditions may be considered an indication for nutritional intervention in patients undergoing chemotherapy for ovarian cancer: (1) pre-existing malnutrition or nutritional risk; (2) daily energy intake <60% of requirements for >10 days, or when the patient is not expected to be able to eat for more than 7 days; and (3) in patients who have recently lost >5% of their body weight due to inadequate nutritional intake [3, 7, 8] | 5.0 | 0 |
| **3.2** **Nutrition education and dietary guidance** | 5.0 | 0 |
| **3.2.1** As the preferred modality, individualised guidance and education is provided throughout the consultation [2, 7, 8] | 5.0 | 0 |
| **3.2.2** Patients with malnutrition or at risk of malnutrition do not use diets that restrict energy intake [1, 2] | 4.50±0.52 | 0.116 |
| **3.2.3** Adopt a Mediterranean diet with at least half plant foods, such as vegetables, fruits, and whole grains, and half or less animal foods, with fish and poultry being the best choices, and reduce saturated fats and red meat intake [1, 2, 9] | 4.67±0.49 | 0.105 |
| **3.2.4** Limit intake of beverages with added sugars or fats, ultra-processed foods, and grilled/marinated animal foods [1, 10] | 4.33±0.49 | 0.114 |
| **3.2.5** In cases of micronutrient deficiencies, dietary fortification with partial nutrient supplementation may be considered while medical treatment is being sought [1] | 4.50±0.52 | 0.116 |
| **3.2.6** Avoid alcohol [1, 9] | 4.33±0.49 | 0.114 |
| **3.2.7** Eat small, frequent meals, at times when the patient is more comfortable [6] | 4.33±0.49 | 0.114 |
| **3.3 Oral nutritional supplements (ONS)** | 5.0 | 0 |
| **3.3.1** ONS are recommended when oral intake does not meet nutritional requirements after nutritional education [1-3, 7, 8] | 5.0 | 0 |
| **3.3.2** Follow the principle of individualisation and choose the type of ONS and intake according to the patient's actual situation [7] | 5.0 | 0 |
| **3.4** **Enteral nutrition (EN)** | 5.0 | 0 |
| **3.4.1** If ONS still fails to meet nutritional needs and the intestinal function is normal, EN should be considered first [1-3, 7] | 5.0 | 0 |
| **3.4.2** Select the appropriate route of EN according to the individual patient's condition [3, 7, 8] | 4.42±0.52 | 0.117 |
| **3.4.3** Use of tumour-specific EN preparations containing complex amino acids and omega-3 PUFA [3, 7] | 4.33±0.49 | 0.114 |
| **3.5** **Parenteral Nutrition (PN)** | 4.92±0.29 | 0.059 |
| **3.5.1** PN is not routinely administered [3, 8] | 5.0 | 0 |
| **3.5.2** Supplemental parenteral nutrition may be given in combination if normal nutritional requirements cannot be obtained by the EN route for a prolonged period of time (≥10 days); total parenteral nutrition may be considered in chemotherapy patients with contraindications to enteral nutrition (e.g., gastrointestinal obstruction, disorders of intestinal metabolic energy supply, etc.) or EN intolerance [1-3, 7, 8] | 4.42±0.52 | 0.117 |
| **3.5.3** Use of PN preparations containing a full range of amino acids and medium/long-chain fat emulsions, especially in patients with combined hepatic dysfunction [3, 8] | 4.50±0.52 | 0.116 |
| **3.5.4** Fatty milks should be used according to the patient's lipid metabolism [3, 7, 8] | 4.58±0.52 | 0.112 |
| **3.5.5** The use of PN should be reduced by gradually increasing EN after the patient's nutritional status and intestinal function have recovered [7] | 5.0 | 0 |
| **4 Symptom management** |  |  |
| **4.1 Prevention of refeeding syndrome** In patients with a significant reduction in food intake lasting more than 5 days, it is recommended that nutritional intake be increased slowly over the first few days, and that vital signs, water electrolytes and micronutrients be monitored closely [2, 7, 8] | 4.25±0.45 | 0.106 |
| **4.2 Loss of appetite** Cortisol-based hormones and progestins should be used under the supervision of the physician to increase the patient's appetite, but attention should be paid to the side effects of the drugs [2, 3] | 4.33±0.49 | 0.114 |
| **4.3 Chemotherapy-induced nausea and vomiting (CINV)** | 5.0 | 0 |
| **4.3.1** The whole management of CINV before, during and after treatment [11, 12]: (1) before treatment: assess the risk factors of CINV in order to individually predict the probability of the occurrence of CINV in patients, and carry out the publicity and education on the adverse effects of antiemetic drugs; (2) during treatment: strengthen the communication with the patients, especially after the emergence of adverse reactions to give symptomatic treatment; (3) after treatment: for the patients who are likely to experience anticipatory nausea and vomiting, provide the patients with the most appropriate treatment for them. (3) After treatment: for patients who may experience anticipatory nausea and vomiting, provide them with more knowledge about CINV and strengthen their psychological counselling. | 5.0 | 0 |
| **4.3.2** Follow-up, recording and dynamic assessment of patients: follow-up methods include telephone and micro-letter; follow-up time is recommended from the beginning of chemotherapy to the 5th day after chemotherapy; follow-up content includes the frequency and degree of nausea and vomiting, physical condition, and drug intake [12] | 5.0 | 0 |
| **4.4 Intestinal obstruction** |  |  |
| **4.4.1** General treatment: abstinence from drinking and fasting, gastrointestinal decompression and the use of cortisol hormones, antiemetic drugs or antisecretory drugs under the supervision of physicians to control nausea, vomiting and other symptoms caused by intestinal obstruction [4, 7, 8] | 5.0 | 0 |
| **4.4.2** Implement total parenteral nutritional support during obstruction and closely monitor water electrolytes, acid-base balance and other biochemical indicators [4, 7, 8] | 5.0 | 0 |
| **4.4.3** When the obstruction is lifted and gastrointestinal function is restored to the point of being able to eat, feed gradually and eventually replace PN [4, 7, 8] | 5.0 | 0 |
| **4.5** **Early satiety** After diagnosis and treatment of constipation, use gastric stimulant medications under the supervision of a physician, but be aware of the potential effects of the medications [2] | 4.33±0.49 | 0.114 |
| **4.6 Diarrhoea** In patients with chemotherapy-induced mucositis and diarrhoea, consider the use of enteral immunomodulatory formulas; however, when the patient is experiencing a severe stressful situation such as a serious infection, the application of immunomodulatory formulas should be based on the guidelines related to critical illness [3, 8] | 5.0 | 0 |
| **4.7 Weight** | 5.0 | 0 |
| **4.7.1** Long-chain n-3 fatty acids or fish oil can be supplemented to stabilise or improve body weight in patients receiving chemotherapy and experiencing weight loss [2, 3, 7, 8] | 4.58±0.52 | 0.112 |
| **4.7.2** Maintain a BMI of 18.5 to 24.9 kg/m2[2, 4, 9] | 4.75±0.45 | 0.095 |
| **5 Physical activity** | 4.42±0.52 | 0.117 |
| **5.1** It is recommended that ovarian cancer patients maintain or increase their level of physical activity during chemotherapy [1, 2, 6, 7] | 4.17±0.39 | 0.093 |
| **5.2 Exercise content** | 4.25±0.45 | 0.106 |
| **5.2.1** Appropriate exercise modes and exercise volume should be selected according to the individualization of patients [6, 7] | 4.33±0.49 | 0.114 |
| **5.2.2** Individualized resistance exercise based on aerobic exercise is recommended [1, 2, 6, 7, 9, 13] | 4.25±0.45 | 0.106 |
| **5.2.3** Exercise duration and intensity should be gradual, 10-60 minutes per exercise session, 3-5 times per week [7, 13] | 4.17±0.39 | 0.093 |
| **5.2.4** Avoidance of exercise-related injuries during exercise [1, 7] | 4.17±0.39 | 0.093 |
| **5.3** Optimal state of exercise Slight sweating of the whole body without feeling tired is appropriate [1, 7] | 4.25±0.45 | 0.106 |
| **6 Evaluation and Follow-up** | 5.0 | 0 |
| **6.1 Efficacy evaluation** | 4.58±0.52 | 0.112 |
| **6.1.1** Nutritional evaluation of the patients was conducted regularly after nutritional intervention [7, 8] | 5.0 | 0 |
| **6.1.2** Nutritional evaluation indexes and time [8]: (1) Rapid change indexes: blood routine, inflammatory parameters, liver function, nutritional package (albumin, etc.), etc., 1 to 2 times a week. (2) Medium-rate change indicators: anthropometric parameters measurements, physical fitness assessment, etc., assessed every 4 to 12 weeks. (3) Slow change indicators: survival time, assessed once a year | 4.42±0.52 | 0.117 |
| **6.2 Follow-up** | 5.0 | 0 |
| **6.2.1** Patients should receive regular nutritional follow-up [7, 8] | 4.17±0.72 | 0.172 |
| **6.2.2** An individualized nutritional follow-up program should be developed according to the patient's specific situation [8] | 5.0 | 0 |

The two enrolled master's degree in nursing and three specialist nurses were responsible for literature search, quality evaluation of the literature, evidence extraction and integration, preparation of the expert correspondence questionnaire and analysis of the correspondence data. The nurse manager was responsible for quality control. We initially formulated the first draft of a multidisciplinary full-course nutritional management program for OC patients undergoing chemotherapy for filling the health education column of WeChat applets through an evidence-based search of domestic and international literature. Afterwards, we used the Delphi method to consult with 12 experts and adjusted the indicators according to the experts’ opinions to form the final draft of the protocol. The return rate of the questionnaire for the 2 rounds of expert consultation was 100%, which indicated that the experts were highly motivated. The degree of expert authority is expressed by the coefficient of expert authority (Cr), which is determined by two factors: the basis of expert judgement on the issue and the expert’s familiarity with the issue. The basis of expert’s judgement on the issue mainly includes four dimensions: practical experience, theoretical analysis, reference to domestic and foreign literature, intuitive feeling. Each dimension is divided into three degrees: large, medium and small; different dimensions of large, medium and small different levels of the assignment are as follows: theoretical analysis (0.3, 0.2, 0.1), practical experience (0.5, 0.4, 0.3), reference to domestic and foreign literature (0.1, 0.1, 0.1), intuitive feeling (0.1, 0.1, 0.1). The basis of judgement, denoted by Ca, is the sum of the scores on the four dimensions mentioned above. Experts' familiarity with the issue and the assignment of points are divided into 5 levels: very familiar (1), more familiar (0.8), generally familiar (0.5), not too familiar (0.2), very unfamiliar (0). The degree of familiarity is expressed by Cs, calculate the mean value of the degree of familiarity of all experts. Cr = [Ca (mean) + Cs (mean)]/2. The general Cr ≥ 0.7 is considered to be reliable. The coefficient of authority is 0.933 (The detailed calculation process is shown in the table below), which means that the experts have a high degree of authority. Kendall’s Coefficient of Coordination (Kendall's W) is a statistic that calculates the degree of correlation of multiple ranked variables and is used as an indicator to test the consistency of the results of the experts' ratings of the indicators. The value of W ranges from 0 to 1, with the closer it is to 1 indicating that the higher the degree of coordination of the experts. This can generally be achieved through SPSS, and the Kendall coefficient can be calculated through the following steps: Analysis - Nonparametric test - K relevant samples test. The Kendall harmony coefficients were 0.257 and 0.520 (P<0.001), suggesting a good degree of coordination among experts. The final nutritional management program contained 6 primary indicators, 24 secondary indicators and 34 tertiary indicators. Details of the program are shown in Supplementary Table 1.

The expert questionnaire requires experts to rate the importance of each indicator of the program, and the importance rating is based on the Likert scale, with ‘unimportant’, ‘not very important’, ‘generally important’, ‘more important’ and ‘very important’ scoring 1 to 5 points respectively. The average score of all the experts for each indicator is the average importance score of the indicator. The coefficient of variation (Cv) indicates the degree of coordination among experts on the relative importance of an indicator, the reasonableness of the calculation formula and the operability of the collection method, and the smaller the coefficient, the higher the degree of coordination among experts. The coefficient of variation is generally considered to be less than 0.25. Cv = mean/standard deviation（$\bar{x}$/s）.

|  | The basis of expert judgement | | | |  | The expert's familiarity with the issue |
| --- | --- | --- | --- | --- | --- | --- |
| Experts | Theoretical analysis | Practical experience | Reference to domestic and foreign literature | Intuitive feeling | Total score for the basis of expert judgement on the issue | Score for expert familiarity with the issue |
| 1 | 0.2 | 0.5 | 0.1 | 0.1 | 0.9 | 1 |
| 2 | 0.2 | 0.5 | 0.1 | 0.1 | 0.9 | 1 |
| 3 | 0.3 | 0.5 | 0.1 | 0.1 | 1 | 1 |
| 4 | 0.3 | 0.5 | 0.1 | 0.1 | 1 | 0.8 |
| 5 | 0.2 | 0.5 | 0.1 | 0.1 | 0.9 | 1 |
| 6 | 0.2 | 0.4 | 0.1 | 0.1 | 0.8 | 0.8 |
| 7 | 0.3 | 0.5 | 0.1 | 0.1 | 1 | 1 |
| 8 | 0.3 | 0.5 | 0.1 | 0.1 | 1 | 1 |
| 9 | 0.2 | 0.5 | 0.1 | 0.1 | 0.9 | 1 |
| 10 | 0.2 | 0.5 | 0.1 | 0.1 | 0.9 | 1 |
| 11 | 0.2 | 0.4 | 0.1 | 0.1 | 0.8 | 0.8 |
| 12 | 0.3 | 0.4 | 0.1 | 0.1 | 0.9 | 1 |
| Mean |  |  |  |  | 0.916667 | 0.95 |
| Cr=[Ca（Mean）+ Cs（Mean）]/2=（0.91667+0.95）/2=0.933 | | | | | | |

**References**

[1] CL Rock, CA Thomson, KR Sullivan, CL Howe, LH Kushi, BJ Caan, ML Neuhouser, EV Bandera, Y Wang, K Robien, KM Basen-Engquist, JC Brown, KS Courneya, TE Crane, DO Garcia, BL Grant, KK Hamilton, SJ Hartman, SA Kenfield, ME Martinez, JA Meyerhardt, L Nekhlyudov, L Overholser, AV Patel, BM Pinto, ME Platek, E Rees-Punia, CK Spees, SM Gapstur, ML Mccullough. American Cancer Society nutrition and physical activity guideline for cancer survivors [J]. CA Cancer J Clin, 2022, 72(3): 230-62.

[2] M Muscaritoli, J Arends, P Bachmann, V Baracos, N Barthelemy, H Bertz, F Bozzetti, E Hütterer, E Isenring, S Kaasa, Z Krznaric, B Laird, M Larsson, A Laviano, S Mühlebach, L Oldervoll, P Ravasco, TS Solheim, F Strasser, M De Van Der Schueren, JC Preiser, SC Bischoff. ESPEN practical guideline: Clinical Nutrition in cancer [J]. Clin Nutr, 2021, 40(5): 2898-913.

[3] 化疗患者营养治疗指南 [J]. 肿瘤代谢与营养电子杂志, 2016, 3(03): 158-63.

[4] 韩娜, 石汉平. 卵巢癌患者的营养治疗专家共识 [J]. 肿瘤代谢与营养电子杂志, 2020, 7(04): 418-20.

[5] B Alderman, L Allan, K Amano, C Bouleuc, M Davis, S Lister-Flynn, S Mukhopadhyay, A Davies. Multinational Association of Supportive Care in Cancer (MASCC) expert opinion/guidance on the use of clinically assisted nutrition in patients with advanced cancer [J]. Support Care Cancer, 2022, 30(4): 2983-92.

[6] N Kiss, J Loeliger, M Findlay, E Isenring, BJ Baguley, A Boltong, A Butler, I Deftereos, M Eisenhuth, SF Fraser, R Fichera, H Griffin, S Hayes, E Jeffery, C Johnson, C Lomma, B Van Der Meij, C Mcintyre, T Nicholls, L Pugliano, T Skinner, J Stewart, J Bauer. Clinical Oncology Society of Australia: Position statement on cancer-related malnutrition and sarcopenia [J]. Nutr Diet, 2020, 77(4): 416-25.

[7] 中国临床肿瘤学会指南工作委员会组织编写. 中国临床肿瘤学会（CSCO）恶性肿瘤患者营养治疗指南 [M]. 2019.

[8] 中国抗癌协会肿瘤营养专业委员会，中华医学会肠外肠内营养学分会组织编写. 中国肿瘤营养治疗指南 2020 [M]. 2020.

[9] R De Las Peñas, M Majem, J Perez-Altozano, JA Virizuela, E Cancer, P Diz, O Donnay, A Hurtado, P Jimenez-Fonseca, MJ Ocon. SEOM clinical guidelines on nutrition in cancer patients (2018) [J]. Clin Transl Oncol, 2019, 21(1): 87-93.

[10] E Gliwska, D Guzek, Z Przekop, J Sobocki, D Głąbska. Quality of Life of Cancer Patients Receiving Enteral Nutrition: A Systematic Review of Randomized Controlled Trials [J]. Nutrients, 2021, 13(12):

[11] F Roila, A Molassiotis, J Herrstedt, M Aapro, RJ Gralla, E Bruera, RA Clark-Snow, LL Dupuis, LH Einhorn, P Feyer, PJ Hesketh, K Jordan, I Olver, BL Rapoport, J Roscoe, CH Ruhlmann, D Walsh, D Warr, M Van Der Wetering. 2016 MASCC and ESMO guideline update for the prevention of chemotherapy- and radiotherapy-induced nausea and vomiting and of nausea and vomiting in advanced cancer patients [J]. Ann Oncol, 2016, 27(suppl 5): v119-v33.

[12] 上海市抗癌协会癌症康复与姑息专业委员会. 化疗所致恶心呕吐全程管理上海专家共识(2018年版) [J]. 中国癌症杂志, 2018, 28(12): 946-60.

[13] T Maurer, MH Belau, J Von Grundherr, Z Schlemmer, S Patra, H Becher, KH Schulz, BC Zyriax, B Schmalfeldt, J Chang-Claude. Randomised controlled trial testing the feasibility of an exercise and nutrition intervention for patients with ovarian cancer during and after first-line chemotherapy (BENITA-study) [J]. BMJ Open, 2022, 12(2): e054091.
